# Medical education in the digital age: Digital whole slide imaging as an e-learning tool

**DOI:** 10.4103/2153-3539.68331

**Published:** 2010-08-10

**Authors:** Kirk Foster

**Affiliations:** Department of Pathology and Microbiology, University of Nebraska Medical Center, Omaha, NE, USA

## INTRODUCTION

The visual aids used in teaching and lecturing to students have clearly evolved over the last several decades. Illustrations on paper were replaced by carousels full of photographic slides, which in turn have been supplanted by Powerpoint™ presentations. At an arguably much slower pace, the method of teaching microscopic anatomy to students of the healthcare professions is also changing. For the majority of medical students graduating before 2000, the microscope and accompanying boxes of glass slides were the standard tools for learning both histology and pathology. Studying for exams also included reviewing kodachromes showing static fields-of-view at predetermined magnifications. Both of these methods of learning required the student to be physically present on campus. Pathology residents, when not learning at the microscope with the attending pathologist, must rely on teaching sets of glass slides that cannot leave the department. Group learning must either be done at a multiheaded microscope (which many times has fewer heads than people present) or by use of video technology attached to the microscope. With the advent of digital whole slide imaging (WSI) over the past several years, there is an opportunity to revolutionize the way teaching and learning are done for all students of medicine including doctors, nurses, medical technicians, histology technicians, cytology technicians, and others.

### Comparison of Digital Whole Slide Images with Glass Slides

There are many advantages to using WSI when compared with traditional glass slides [Table 1]. Digital images can be standardized; all students will study the exact same tissue section. Sections on glass slides are inherently variable in quality and content.[1] Although admittedly subtle in some cases, these variabilities can be eliminated with digital imaging. The quality of the image can also be indefinitely maintained compared to glass slides that are vulnerable to fading, breaking, and vanishing.[2] One very helpful aspect of digital images is the use of a thumbnail image. As students are examining the image at higher magnification, they can always refer to the thumbnail for orientation.[3] This obviously is impossible with glass slides. Glass slides cannot be easily annotated with any precision, relying on relatively crude “dotting” for the purposes of highlighting a certain area of the slide. Digital images can have multiple annotations including arrows, circles, text, etc. placed exactly where needed. Portability is another benefit of using digital images. The use of microscopes obligates a student to remain on campus in order to study, review, etc. With WSI loaded onto a web-based server, study can occur wherever and whenever the student wishes.[2] WSI also makes simultaneous viewing of a particular field-of-view possible; a major advantage over glass slides. Viewing the image on a common computer monitor encourages discussion and collaboration between classmates.[1] Finally, storage of WSI becomes a matter of having enough server memory. As server space becomes less and less expensive, the cost and effort of storing and maintaining both the microscopes and glass slide sets will become comparatively more burdensome.[3] Some disadvantages that have become apparent with WSI include the dependence of the image quality on monitor resolution and the challenge of scanning tissue sections that have artifacts such as folds, etc.[3] Judicious choice of an acceptable non-cost prohibitive monitor is essential for an accurate image. As digital scanners become “smarter”, the flaws in a slide will likely become less of an issue.

**Table 1:** Benefits of digital slide imaging

Digital WSI	Glass slides
Ability to standardize image	Section-to-section variability
Indefinite maintenance of image quality	Fading, breakage, etc.
Thumbnail image allows student orientation	No overview image available
Annotation of images with text, arrows, etc.	No annotation possible (other than crude dotting, etc.)
Portability (time and location)	Student tied to microscope
Collaboration between students easier	Collaboration limited
Easier storage (requires server space)	Cumbersome storage of both microscopes and slides

### Educational Applications of Digital Whole Slide Imaging

The educational applications for this technology are growing. Histology and pathology laboratories and small group study sessions are the early opportunities for medical student use either in a formal computer laboratory or via personal laptops. For those students choosing a pathology residency, WSI can be used for didactic lectures and unknown conferences. Independent study can be encouraged and facilitated with the use of organ-system-based teaching sets, of both neoplastic and non-neoplastic diseases. Diagnostic skills can also be assessed with WSI exams. One academic center looked at the reliability of WSI for measuring resident competency. Using a set of 20 questions based upon 20 whole slide images, junior and senior residents were tested. They determined that the correlation between exam score and months of training was high, the correlation with the resident in-service examination (RISE) was not quite as high, and that the exam could reliably discriminate between junior and senior residents.[4] WSI can also be used for teaching residents or fellows in clinical fields. For pathologists in either academic or non-academic medical centers, WSI can be implemented into both teaching and working interdepartmental conferences such as tumor boards and biopsy conferences (renal, liver, transplant, etc.). For the established pathologist, WSI has become a convenient method of acquiring continuing medical education credits offered through national pathology organization websites. Additional uses for WSI including the creation of electronic books will certainly evolve over time.

### The University of Nebraska Medical Center Experience

**Figure 1:**
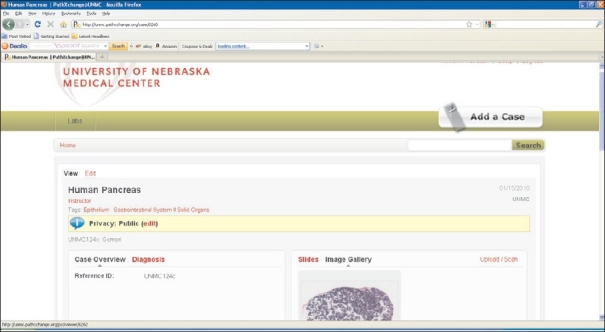
Case editing page. Slide images can be uploaded to the image gallery from this page

**Figure 2:**
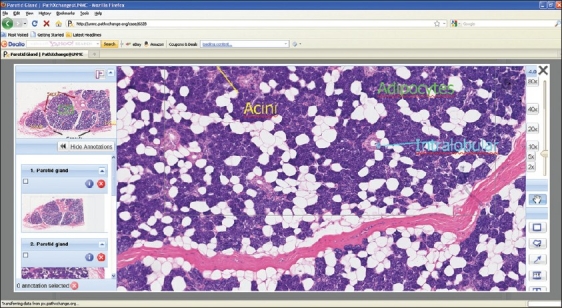
Viewer showing image with annotations

The University of Nebraska Medical Center (UNMC) has chosen to partner with Pathxchange™ for the creation of both first year histology and second year pathology medical student laboratories. Pathxchange™ is a vendor neutral website that allows for online sharing of whole slide images and has the ability to define user groups and communities based upon participants’ desired privacy settings. In addition to whole slide images other static images in a variety of file formats can be uploaded to Pathxchange™. Importantly, all images are anonymized as slide labels bearing case numbers, and patient names are not uploaded with the image. Once images are uploaded, they can be annotated. A microsite has been created within Pathxchange™ for UNMC. By design, this microsite will be accessible only to UNMC medical students and pathology residents. UNMC faculty will have authoring rights to generate either collections of histology images for first year medical students or case scenarios for second year pathology students. Case scenarios can be authored by uploading whole slide images of pathologic lesions and including associated pertinent clinical history, radiographic images, gross pathology images, and any informative ancillary studies such as cytogenetics, etc. [Figure 1]. Students will have the option to either use the annotations, or for those students wanting to self-test their recall of the microanatomy/pathology, it will also be possible to “hide” the annotations [Figure 2]. The images/cases will be available for the students at all times via their Internet browser.

## CONCLUSION

Digital whole slide imaging is a powerful educational tool that effectively replaces the traditional standard methods of teaching and learning both histology and pathology. It provides mobility and convenience to medical students. While there are still some medical schools using microscopes and glass slides, the number of schools doing so continues to decrease. The use of this technology can also easily be applied to other types of students in the medical field.
